# Focused and Sustained Attention Is Modified by a Goal-Based Rehabilitation in Parkinsonian Patients

**DOI:** 10.3389/fnbeh.2017.00056

**Published:** 2017-03-31

**Authors:** Davide Ferrazzoli, Paola Ortelli, Roberto Maestri, Rossana Bera, Roberto Gargantini, Grazia Palamara, Marianna Zarucchi, Nir Giladi, Giuseppe Frazzitta

**Affiliations:** ^1^Department of Parkinson’s disease, Movement Disorders and Brain Injury Rehabilitation, Moriggia Pelascini HospitalComo, Italy; ^2^Department of Biomedical Engineering, Istituti Clinici Scientifici Maugeri Spa Società Benefit, IRCCS MontescanoPavia, Italy; ^3^Movement Disorders Unit, Neurological Institute, Tel-Aviv Medical Centre, Sagol School for Neuroscience, Sackler School of Medicine, Tel-Aviv UniversityTel-Aviv, Israel

**Keywords:** Parkinson’s disease, reaction times, rehabilitation, attention, executive functions

## Abstract

Rehabilitation for patients with Parkinson’s disease (PD) is based on cognitive strategies that exploit attention. Parkinsonians exhibit impairments in divided attention and interference control. Nevertheless, the effectiveness of specific rehabilitation treatments based on attention suggests that other attentional functions are preserved. Data about attention are conflicting in PD, and it is not clear whether rehabilitative treatments that entail attentional strategies affect attention itself. Reaction times (RTs) represent an instrument to explore attention and investigate whether changes in attentional performances parallel rehabilitation induced-gains. RTs of 103 parkinsonian patients in “on” state, without cognitive deficits, were compared with those of a population of 34 healthy controls. We studied those attentional networks that subtend the use of cognitive strategies in motor rehabilitation: alertness and focused and sustained attention, which is a component of the executive system. We used visual and auditory RTs to evaluate alertness and multiple choices RTs (MC RTs) to explore focused and sustained attention. Parkinsonian patients underwent these tasks before and after a 4-week multidisciplinary, intensive and goal-based rehabilitation treatment (MIRT). Unified Parkinson’s Disease Rating Scale (UPDRS) III and Timed Up and Go test (TUG) were assessed at the enrollment and at the end of MIRT to evaluate the motor-functional effectiveness of treatment. We did not find differences in RTs between parkinsonian patients and controls. Further, we found that improvements in motor-functional outcome measures after MIRT (*p* < 0.0001) paralleled a reduction in MC RTs (*p* = 0.014). No changes were found for visual and auditory RTs. Correlation analysis revealed no association between changes in MC RTs and improvements in UPDRS-III and TUG. These findings indicate that alertness, as well as focused and sustained attention, are preserved in “on” state. This explains why Parkinsonians benefit from a goal-based rehabilitation that entails the use of attention. The reduction in MC RTs suggests a positive effect of MIRT on the executive component of attention and indicates that this type of rehabilitation provides benefits by exploiting executive functions. This ensues from different training approaches aimed at bypassing the dysfunctional basal ganglia circuit, allowing the voluntary execution of the defective movements. These data suggest that the effectiveness of a motor rehabilitation tailored for PD lies on cognitive engagement.

## Introduction

In Parkinson’s disease (PD) the loss of the physiological dopaminergic modulation transforms the basal ganglia into a disruptive filter (Beeler et al., 2013) that impairs the ability to express habitual-automatic actions (Redgrave et al., 2010). Rehabilitation has been proposed as an effective and complementary approach for the treatment of PD (Goodwin et al., 2008; Keus et al., 2009; Frazzitta et al., 2010, 2012, 2015b). The great value of rehabilitation lies in the possibility of treating those disabling PD disturbances, such as balance dysfunctions, postural instability and freezing of gait, that do not respond to the standard medical or surgical treatments.

Even if implicit learning is defective in parkinsonian subjects, motor learning is feasible in PD (Nieuwboer et al., 2009). In this regard, it has been demonstrated that specific rehabilitation techniques provide benefits by using explicit cognitive strategies (Nieuwboer et al., 2009). These are bottom-up strategies, which use external cues, and/or top-down strategies, which exploit feedbacks or verbal instructions. The application of these explicit cognitive strategies requires the use of attention (Morris et al., 2010) and activates the goal-directed control system, bypassing the dysfunctional habitual, sensorimotor basal ganglia circuit (Morris, 2006; Morris et al., 2009; Redgrave et al., 2010). Consequently, while it is known that functions such as the divided attention and the interference control might be impaired in PD (Sharpe, 1996; Wylie et al., 2009), the existing evidences about the effectiveness of rehabilitation approaches based on explicit learning strategies (Nieuwboer et al., 2009; Morris et al., 2010) suggest that other attentional functions are preserved or, at least, exploitable in PD patients.

Therefore, it is important to understand which are the attentional functions subtending the use of explicit cognitive strategies in motor rehabilitation and that could be used in the clinical setting in order to develop even more effective rehabilitative protocols for patients with PD. At the same time it is not clear whether rehabilitative treatments that entail attentional strategies can affect attention itself in PD. This issue appears to be really important in light of recent findings showing improvements in cognitive performances after specific motor trainings (David et al., 2015; Manenti et al., 2016).

Petersen and Posner (2012) theorized that the attention is divisible in different sub-functions and that every single sub-function is related to specific anatomical and functional structures. In particular, there are three sub-functions, each representing a different set of attentional processes: alert, orienting and executive systems. Therefore, by administering specific reaction times (RTs) tasks, it is possible to explore the three different attentional networks. RTs measurement represents a valid instrument to assess the attentive resources: it is defined as the elapsed time between the presentation of a sensory stimulus and the subsequent behavioral response and it is considered as an index of attention (Jensen, 2006). To date, behavioral data about RTs in parkinsonian patients are conflicting. This is probably due to the subtype of the attentional network under investigation (Dujardin et al., 2013), the clinical state (“on” vs. “off”) of the patients (Pullman et al., 1990) and their executive performances (Berry et al., 1999). Dujardin et al. (2013) found that RTs exploring alert are comparable with those of a control population of healthy subjects, while the tasks exploring the executive component of attention are slower. Pullman et al. (1990) tried to overcome the problem of medications investigating choice and simple RTs in relation to circulating levels of levodopa. These authors found that the choice RTs in PD were not different from normal and were only slightly longer than simple RTs when patients were medicated. However, in “off” clinical state, the choice RTs were sufficiently delayed to be significantly different from normal, while the simple RTs were unresponsive to changes in concentration of levodopa. Berry et al. (1999) distinguished PD patients on the basis of their performances in executive tasks. These authors found that performances of the patients non-frontally impaired were the same of the controls. By contrast, the “frontally-impaired” parkinsonian subjects responded significantly more slowly than the control.

It is conceivable that the use of explicit cognitive strategies in motor rehabilitation in PD is subtended by the alertness and by the focused and sustained attention. These attentional networks probably drive the behavioral-motor responses in PD patients undergoing a motor rehabilitation program based on cognitive strategies (such as cues, feedbacks and verbal instructions). Alertness is the state of active attention by high sensory awareness; focused and sustained attention is a component of the executive system (Sturm and Willmes, 2001) and represents the ability to focus cognitive activity on specific stimuli or on one specific task over time. A deficit in alertness makes the patients unable to produce fast responses, while a deficit in the executive system determines disturbances in focusing and maintaining attention in a specific target and/or in a specific skill over time (Sturm and Willmes, 2001). Therefore, in relation to the previous findings about RTs in PD patients and in order to better understand whether these above-mentioned attentional functions are preserved in parkinsonian patients, we have compared RTs of parkinsonian patients in “on” state, without cognitive deficits, with those of a population of healthy controls.

According to the Petersen and Posner’s (2012) theory we used visual (V RTs) and auditory RTs (A RTs) to evaluate the alertness and multiple choices RTs (MC RTs) to explore the executive component of attention. Since parkinsonian patients develop over time deficits in spatial abilities (Cronin-Golomb and Amick, 2001) and in spatial cognition (Fimm et al., 2001), we study alertness by using not only visual stimuli but also auditory stimuli. Further, in order to investigate whether a motor rehabilitation program, based on explicit learning strategies, affects the attentional performances itself, PD patients underwent these attentive tasks before and after a 4-week multidisciplinary intensive rehabilitation treatment (MIRT).

## Materials and Methods

### Study Population

Between January and May 2016, we enrolled at the Department of Parkinson’s disease, Movement Disorders and Brain Injury Rehabilitation (“Moriggia-Pelascini” Hospital, Gravedona ed Uniti—Como, Italy), 103 PD patients hospitalized for a 4-week MIRT (Frazzitta et al., 2010, 2012, 2015b). Parkinsonian patients were diagnosed according to the UK Brain Bank criteria (Hughes et al., 1992) and were evaluated by a neurologist with experience in movement disorders.

The inclusion criteria were: (i) stage 2.5–3 according to the Hoehn and Yahr scale (H&Y); (ii) stable pharmacological treatment for the last 6 weeks before the enrollment and during the hospitalization; (iii) Mini Mental State Examination (MMSE) ≥ 24 (Folstein et al., 1975); and (iv) no evidences of dysexecutive syndrome (Godefroy et al., 2010).

Exclusion criteria were: (i) any focal brain lesion detected in brain imaging studies (CT or MRI) performed in the previous 12 months; (ii) drug-induced dyskinesias; (iii) disturbing resting and/or action tremor, corresponding to scores 2–4 in the specific items of Unified Parkinson’s Disease Rating Scale (UPDRS) III; (iv) behavioral disturbances (evaluated with Neuropsychiatric Inventory); and (v) visual and auditory dysfunctions according to the general clinical evaluation and medical history.

Thirty-four healthy subjects matched for age, sex and years of education served as controls.

The study design and protocol were approved by the local Scientific Committee (“Moriggia-Pelascini” Hospital, Gravedona ed Uniti—Como, Italy) and were in accordance with the code of Ethics of the World Medical Association (Declaration of Helsinki, 1967). A complete explanation of the study protocol was provided and written informed consent was obtained from all participants before their participation in the study. This trial was registered with ClinicalTrials.gov, NCT02727257.

### Neuropsychological Assessment and Attentional Tasks

Patients underwent attentive tasks at 9 AM, during the medication “on” state, at the enrollment and at the end of MIRT. Healthy controls were evaluated at the same time of the day. All subjects were tested in a laboratory setting, with constant lighting condition (artificial light) and without auditory interferences.

Neuropsychological assessment included the MMSE (Folstein et al., 1975; Magni et al., 1996) and the frontal assessment battery (FAB; Dubois et al., 2000; Appollonio et al., 2005).

**Figure 1:**
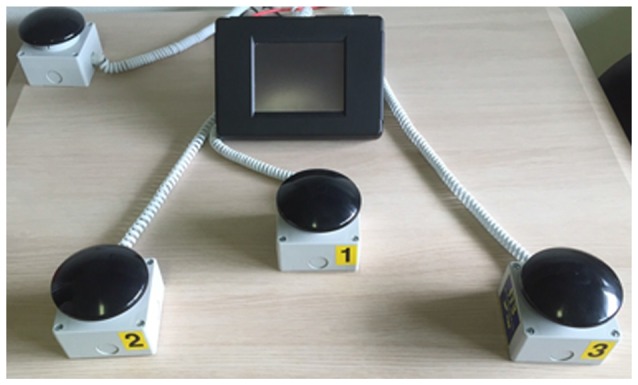
**Device used to assess attention.** System used to assess attention by the evaluation of the performance in a computer-controlled reaction times (RTs) paradigm (ITB Sport Reflection, F.M. Automazione S.r.l., Brescia, Italia).

Attention was assessed by the evaluation of the performance in a randomized computer-controlled RTs paradigm designed to measure different attentional components (ITB Sport Reflection, F.M. Automazione S.r.l., Brescia, Italia; see Figure 1).

Subjects were asked to seat directly in front of a 5.7″ diagonal color monitor (display resolution 320 × RGB × 240; Pixel Pitch 0.36 H × 0.36 V; active area 115.2 W × 86.4 H; outline dimension 144.0 W × 140.6 H × 12.8 T without FPCB tail; color garmut NTSC 58%) at a distance that was comfortable to them. The investigator read the task’s instruction before the starting of the experimental task. For each task, subjects performed one training section in order to become confident with the experiment and avoid the bias related to the learning effect of test-retest. Subjects were instructed to place their preferred hand on a specific position indicated on table with a black line, located at the same distance from the response buttons. Subjects had also to fix the screen and to press, with their preferred hand, the response key (button response) when a target-stimulus appeared. Between the stimuli, the patient had to remain with the hand at rest on the table. Three different attentive tasks were performed: visual RT (V RT) task, auditory RT (A RT) task and MC RT task. The test session lasted 45 min.

#### Visual Reaction Times Task

**Figure 2:**
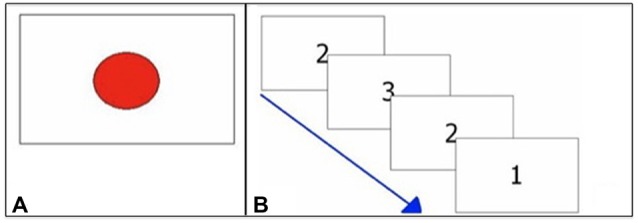
**Exemplifications of attentional tasks. (A)** Visual RTs task (V RTs); **(B)** Multiple choices RTs (MC RTs) task.

The task consisted of 40 trials. In each single trial, the subjects had to press a response button as quickly as possible at the appearance of the target that disappeared after the subject’s response. Target was a red circle (see Figure 2A) that appeared on the center of screen at irregular intervals (1–3 s). The times between the appearance of the target and the subject’s response were recorded. Response times shorter than 250 ms and longer than 1000 ms were deemed to be outliers and were excluded from analysis. The number of RTs excluded from the analysis was recorded. The median value was taken as representative of the central tendency of each subject (Ratcliff, 1993; Ratcliff and Van Dongen, 2011).

#### Auditory Reaction Times Task

The task consisted of 40 trials. In each single trial the subjects had to press a response button as quickly as possible at the presentation of the target that consisted of an acoustic stimulus (intensity of 94 dbA) presented at irregular intervals (1–3 s). The stimulus ended after the subject’s response. The times between the presentation of the target and the subject’s response were recorded. Response times shorter than 250 ms and longer than 1000 ms were deemed to be outliers and were excluded from analysis. The number of RTs excluded from the analysis was recorded. The median value was taken as representative of the central tendency of each subject.

#### Multiple Choices Reaction Times Task

The task consisted of 40 trials. The target was a number (1, 2, 3) whose presentation, on the center of the screen, was randomized. Each number was associated to a different response buttons (see Figure 1). In each single trial subjects had to press as quickly as possible the response button associated with the number that appeared on the screen (see Figure 2B).

The accuracy of responses was evaluated by counting the errors. The times between the presentation of the stimuli and the subject’s response were recorded: RTs shorter than 250 ms and longer than 2000 ms were deemed to be outliers and were excluded. The number of RTs excluded from the analysis was recorded. The median value of valid response times was taken as representative of the central tendency of each subject.

### Rehabilitation Treatment

MIRT is specifically designed for PD patients (Frazzitta et al., 2012, 2013, 2015b). Previous controlled randomized trials showed the effectiveness of MIRT on motor disturbances in PD patients in early and medium stages of disease (Frazzitta et al., 2012, 2015b; Volpe et al., 2014). It is a multidisciplinary, aerobic, intensive and goal-based rehabilitation treatment, aiming at re-learning the dysfunctional movements using motor-cognitive strategies. All the activities included in the protocol are performed with a heart rate reserve comprised between 70% and 80%. MIRT consists of a 4-week physical therapy, in a hospital setting, which entails four daily sessions for 5 days and 1 h of physical exercise on the 6th day. The duration of each session, including recovery periods, is about 1 h. The first session consists of a one-to-one session with a physical therapist and comprises cardiovascular warm-up activities, active and passive exercises to improve the range of motion of all the different joints, stretching of the abdominal muscles, strengthening of paravertebral muscles, postural changes and exercises specifically addressed to improve balance and postural control. The second session exploits the use of different devices to improve gait and balance: a stabilometric platform with visual cues (patients had to follow a pathway on a screen by using a cursor sensitive to their feet movements on the platform), treadmill plus (treadmill training with visual cues and auditory feedbacks; Frazzitta et al., 2009), crossover (Frazzitta et al., 2015a) and cycloergometer with visual cue. The maximum speed of treadmill scrolling is 3.5 Km/h; patients are trained with treadmill no more than 15 min, two times per day. The selection of the devices to adopt and the training parameters setting are defined for each patient in relation to the disease severity. The third is a session of occupational therapy aimed at improving autonomy in activities of daily life. The last session includes 1 h of speech therapy. On the 6th day the patients are trained for 1 h by using the devices. The rehabilitation program is personally tailored for each patient and could include: aquatic therapy (for patients with severe balance and postural disturbances), robotic-assisted walking training (for patients with complex gait disorders), virtual-reality training and psychoeducational groups with the neuropsychologists. A team meeting defines weekly the rehabilitation program for each patient and assesses the benefits.

### Clinical Evaluation and Outcome Measures

A neurologist with experience in movement disorders examined the patients in the morning, 1 h after they had taken the first dopaminergic drug dose, in medication “on” state, both at the beginning and at the end of MIRT. UPDRS III and the Timed Up and Go test (TUG) were assessed in order to investigate the clinical and motor-functional effectiveness of MIRT.

### Statistical Analysis

Shapiro–Wilk statistic, supported by visual inspection, was used to assess the normality of all variables. Descriptive statistics are reported as mean ± SD for continuous variables and frequency (%) for categorical variables. Between-group comparisons (patients with PD vs. controls) were carried out by independent samples *t*-test or Wilcoxon-Mann-Withney U test when appropriate for continuous variables and by the Chi-square test for categorical variables.

Within-group comparisons (post-treatment vs. baseline measurements) were carried out by paired *t*-test or by the Wilcoxon signed rank sum test when appropriate. The association between variables was investigated by Pearson correlation coefficient or by Spearman rank correlation coefficient. A *p*-value < 0.05 was considered statistically significant. When appropriate, false discovery rate was controlled at 5% using the Benjamini-Hochberg method. All analyses were carried out using the SAS/STAT statistical package, release 9.2 (SAS Institute Inc., Cary, NC, USA).

## Results

Formal Shapiro–Wilk test for normal distribution was not fully satisfied by some of the variables considered. Hence, non-parametric tests were used. However, since violations to the normality assumption were not strong, obtained results were checked using also parametric statistics, obtaining very similar results. Demographical, clinical data and baseline RTs for patients and controls are reported in Table 1. Patients and controls were not different for sex, age and years of education but showed higher MMSE and FAB scores. All subjects were right-handed.

**Table 1:** **Demographical, clinical data and baseline reaction times (RTs) for patients and controls**.

Variable	Patients (*N* = 103)	Controls (*N* = 34)	*p*-value*
Age (years)	66.2 ± 9.2	65.4 ± 7.1	0.341
Sex (% Male)	55	53	0.496
Education (years)	10.5 ± 4.3	10.4 ± 4.3	0.841
MMSE	27.3 ± 1.9	28.7 ± 1.3	<0.0001
FAB	14.4 ± 2.6	15.8 ± 1.2	0.007
BDI	6.8 ± 4.1		
H&Y	2.6 ± 0.5		
Levodopa equivalent dose (mg/die)	661.8 ± 328.4		
Disease duration (years)	10.3 ± 5.1		
Total UPDRS	40.3 ± 10.8		
Mean value MC RTs	0.98 ± 0.18	0.93 ± 0.17	0.142
Mean value A RTs	0.29 ± 0.09	0.29 ± 0.09	0.680
Mean value V RTs	0.34 ± 0.07	0.34 ± 0.08	0.584

*p-values are for the comparison Patients vs. Controls, Mann-Whitney U test. Abbreviations: MMSE, Mini Mental State Examination; FAB, Frontal Assessment Battery; BDI, Beck Depression Inventory; H&Y, Hoehn and Yahr staging scale; UPDRS, Unified Parkinson’s Disease Rating Scale; MC RTs, Multiple choices reaction times task; A RTs, Auditory reaction times task; V RTs, Visual reaction times task.

The rate of errors in accomplishing the MC RTs trials was very low for all subjects, ranging from 0 to 3 out of the 40 trials. Globally 94% of evaluations were without errors. A qualitative evaluation of the response was not possible to perform for visual and auditory RTs tasks, since only one stimulus (visual or auditory respectively) was provided and the software was set to exclude response times shorter than 250 ms and longer than 1000 ms.

The number of repeated trials was very low for all subjects. Globally, it was not greater than 5%.

Baseline results for RTs in patients and controls are also reported in Table 1. No significant differences were found in visual, auditory and MC RTs between patients and controls at baseline.

Table 2 reports patients’ scores of UPDRS III, TUG and RTs values at baseline and after MIRT.

**Table 2:** **RTs and clinical-functional data before and after multidisciplinary intensive rehabilitation treatment (MIRT), with percent differences (end of treatment—basal values)**.

Variable	Admission	Discharge	Delta (%)	*p*-value*
UPDRS III (“On” state)	18.6 ± 4.9	13.0 ± 4.6	−27.7 ± 41.8	<0.0001
TUG	11.7 ± 6.2	8.9 ± 4.1	−21.0 ± 12.7	<0.0001
Mean value MC RTs	0.98 ± 0.18	0.94 ± 0.16	−3.2 ± 11.2	0.002
Mean value A RTs	0.29 ± 0.09	0.29 ± 0.10	0.5 ± 21.3	0.373
Mean value V RTs	0.34 ± 0.07	0.33 ± 0.08	−1.3 ± 12.8	0.125

*p-values are for the comparison discharge vs. admission, Wilcoxon signed rank sum test. Abbreviations: UPDRS, Unified Parkinson’s Disease Rating Scale; TUG, Timed up and Go test; MC RTs, Multiple choices reaction times task; A RTs, Auditory reaction times task; V RTs, Visual reaction times task.

Considering RTs, no significant changes were found after rehabilitation for visual and auditory RTs. Conversely, a significant reduction was observed in MC RTs task, with a mean improvement of 40 ms (*p* = 0.0002, significant after Benjamini-Hochberg adjustment). Considering percent changes, the same pattern was observed, with MC RT showing the only significant improvement (*p* = 0.003).

**Figure 3:**
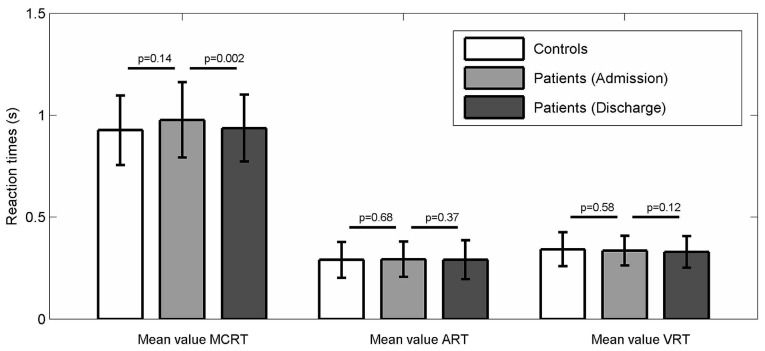
**Patients and Controls performances in the different RTs tasks.** Comparison between controls’ and patients’ performances in auditory, visual and MC RTs at baseline and their variations after multidisciplinary intensive rehabilitation treatment (MIRT).

Figure 3 visually shows the comparison between controls’ and patients’ performances in auditory, visual and MC RTs at baseline and their variations after MIRT.

A significant improvement was observed for UPDRS III and TUG scores (*p* < 0.0001 all) in line with previous findings from our group in controlled randomized trials (Frazzitta et al., 2012, 2015b; Volpe et al., 2014). Correlation analysis (Spearman) revealed no association between changes in MC RTs and improvements in UPDRS III and TUG (*p* = 0.76 and *p* = 0.18, respectively), as well as no association between changes in UPDRS III and TUG (*p* = 0.86).

## Discussion

In this study we did not find any differences in visual, auditory and MC RTs between PD patients in “on”state, without cognitive deficits and healthy controls. Our data suggest that alertness, as well as sustained and focused attention, are preserved in parkinsonian subjects without cognitive deficits, in “on” state. This finding could be explained considering that bradykinesia and bradyphrenia are not necessarily overlapping conditions. While bradykinesia is a distinctive feature of PD, bradyphrenia is a symptom that appears only in particular cognitive conditions, with respect to the pharmacological state or the specific task demand.

The integrity of these attentional functions could partially explain why PD patients benefit from a goal-based and intensive rehabilitation treatment, such as MIRT, which entails the use of attentional, explicit learning strategies.

As expected, we found improvements in motor-functional outcome measures (UPDRS III, TUG), after MIRT. These improvements paralleled a significant reduction in MC RTs. On the contrary, no changes in visual and auditory RTs were found after rehabilitation.

MC RTs explore the focused and sustained attention that is a function of the executive system. Therefore, this selective reduction in MC RTs after MIRT could be related to the specificity of the rehabilitation treatment that provides motor and functional benefits by exploiting the functions of the executive system. This may ensue from different approaches aimed at bypassing the dysfunctional basal ganglia-supplementary motor area circuit deficit (Goldberg, 1985; Morris, 2000) in order to voluntarily express the defective movements: (i) by learning explicit verbal and non-verbal strategies to overcome the motor programming deficits; (ii) by maintaining attention toward a specific goal through the use of cues and feedbacks; (iii) by dual-task training with instructions to equally divide attention between movements (e.g walking) and concurrent cognitive tasks (Fok et al., 2012); and (iv) by exploiting feedbacks in order to improve different motor aspects, such as balance or coordination.

The repetitive and intensive use of these executive-attentional processes leads in turn to a positive treatment-effect on the executive component of attention. Indeed, in this rehabilitative context, the continuous indications given by physiotherapist during the exercises and the use of feedbacks, cues and devices such as treadmill plus, stabilometric platform and cycloergometer, stimulates selective attention processes that enable goal-directed, internally-driven decision.

This finding of a MIRT-induced positive effect on a cognitive component, such as the focused and sustained attention, is in line with previous findings: as a matter of fact, it has been recently demonstrated that resistance exercise improves several non-motor functions in healthy-aging population, including V RTs (Fragala et al., 2014). These evidences from an healthy-aging population suggest that cognition in general could benefit from aerobic exercise. Further, in a recent study, it has been also demonstrated that the presence of cognitive decline does not affect negatively on the motor outcomes of PD patients undergoing a goal-based, intensive and aerobic rehabilitation treatment, such as MIRT (Ferrazzoli et al., 2016). Data from these studies and other findings of improvement in cognitive performances after specific motor trainings (David et al., 2015; Manenti et al., 2016) suggest the possibility that cognition could be influenced from specific and tailored motor rehabilitation programs and lead us to conclude for the existence of a close link between motor and cognitive performances.

With regard to the motor outcomes, we believe that while the improvement in UPDRS III is an index of the beneficial effect of MIRT on motor performances, the improvement in TUG score confirms the involvement of executive functions in the achieving of clinical improvements in rehabilitation. Indeed, TUG is a test that allows evaluating the executive component of action (Morris et al., 2001) and, in line with our data, Manenti et al. (2014) found an improvement in TUG score in PD patients treated with transcranial current stimulation at the level of the dorsolateral prefrontal cortex. The lack of correlation between improvement in motor outcomes (TUG and UPDRS III) and reduction in MC RTs could mean that the motor benefits are achieved by exploiting a combination of both motor and cognitive strategies. One possible explanation for the linkage between the improvements in motor outcomes (particularly in TUG) after MIRT and the reduction in MC RTs could be related to the effect of the treatment on “motor inertia”, which can be understood as the cognitive component of bradykinesia. It is known that PD patients become progressively slower with the increasing of the complexity of the stimuli (Cooper et al., 1994). This slow response is related to two different factors: the deficit in the identification of stimulus and the difficulties in the “internal” representation of motor programming (Cooper et al., 1994). Thus, a specific attention towards the goal of the primary motor task could improve movements in PD (Oliveira et al., 1998). The difficulties in the beginning of movement and the motor programming deficits in PD result from frontal disconnection of the supplementary motor area (Dick et al., 1986; Haslinger et al., 2001). This dysfunction seems to lead to an increased activity of the premotor and parietal cortical areas (Haslinger et al., 2001) and reflects a cognitive compensatory mechanism necessary for the initiation of movement (Rowe et al., 2002). MIRT, involving different training approaches aimed at bypassing the basal ganglia-supplementary motor area circuit deficit (by using cognitive strategies, cues and feedbacks) probably acts by inducing greater attention to action. This could result in a positive effect on the motor executive programming abilities, with a consequent improvement in MC RTs.

Therefore, we could conclude that a goal-based and intensive rehabilitation treatment, such as MIRT, is effective on both motor and cognitive functions.

## Study Limitations

There are a number of limitations to this study that need to be acknowledged. First, we did not perform a detailed neuropsychological assessment. This would allow us to better understand the cognitive profiles of patients in order to evaluate the effect of MIRT on cognition.

Another aspect to take into account is the possibility that our results might be related to a possible placebo effect. However, we found a reduction in MC RTs and no changes in visual and auditory RTs after rehabilitation, thus making this possibility very unlikely. We did not collect follow-up data for this group of patients neither about motor-functional outcomes nor about RTs. Therefore, we cannot say how long the improvements we found last for.

Finally, we did not evaluate the effect of a control procedure similar to MIRT on RTs of healthy control subjects and the effect of other, non goal-based or non-intensive rehabilitative approach different from MIRT in another group of Parkinsonian patients. Further studies are needed for the above-mentioned issues.

## Conclusion

In conclusion, we found that alertness, as well as focused and sustained attention, are preserved in “on” state, in PD patients without cognitive deficits. This allows patients to benefit from rehabilitative treatments that entail the use of attention. Further, we found a significant reduction in MC RTs, over and above the improvement in motor-functional outcome measures after MIRT. This probably means that this type of motor, intensive and goal-based rehabilitation provides motor and functional benefits by exploiting executive functions, leading in turn to a positive treatment-effect on the executive component of attention.

Further studies are needed to better understand how to use the attentive resources of PD patients for rehabilitative purposes and to clarify the effect of tailored rehabilitation programs on attention.

## Author Contributions

DF wrote the text, conceived and designed the experiments, provided substantial contributions to discussion of the content and edited the manuscript before submission; PO wrote the text, conceived and designed the experiments, performed the experiments, provided substantial contributions to discussion of the content and generated figure; RM analyzed the data and did the statistical analysis and generated tables; RB researched data for the article and performed the experiments; RG and MZ researched data for the article, conceived and designed the experiments, performed the experiments; GP wrote the text and provided substantial contributions to discussion of the content; NG did a critical revision and provided substantial contributions to discussion of the content; GF conceived and designed the experiments, analyzed the data, wrote the text and provided substantial contributions to discussion of the content.

## Conflict of Interest Statement

NG serves as a member of the Editorial Board for the Journal of Parkinson’s Disease. He serves as consultant to Teva-Lundbeck, Intec Pharma, NeuroDerm, Arsmon Neuromedical Ltd/Dexel, Monfort and Lysosomal Therapeutic Inc. He received payment for lectures at Teva-Lundbeck, Novartis, UCB, Abviee, Shaier and Genzyme. NG received research support from the Michael J Fox Foundation, the National Parkinson Foundation, the European Union 7th Framework Program and the Israel Science Foundation as well as from Teva NNE program, LTI, and Abviee and CHDI. The funders had no role in study design, data collection and analysis, decision to publish, or preparation of the manuscript. The other authors declare that the research was conducted in the absence of any commercial or financial relationships that could be construed as a potential conflict of interest.
